# CeLLTra: aligning cell names with gene expression via a pathway-informed transformer

**DOI:** 10.1093/bioinformatics/btaf655

**Published:** 2025-12-05

**Authors:** Zhao Li, Zaiyi Zheng, Rongbin Li, Wenbo Chen, Yuntao Yang, Meer A Ali, Jundong Li, W Jim Zheng

**Affiliations:** Department of Bioinformatics & Systems Medicine, McWilliams School of Biomedical Informatics, University of Texas Health Science Center at Houston, Houston, TX 77030, United States; Department of Electrical and Computer Engineering, University of Virginia, Charlottesville, VA 22903, United States; Department of Bioinformatics & Systems Medicine, McWilliams School of Biomedical Informatics, University of Texas Health Science Center at Houston, Houston, TX 77030, United States; Department of Bioinformatics & Systems Medicine, McWilliams School of Biomedical Informatics, University of Texas Health Science Center at Houston, Houston, TX 77030, United States; Department of Genomic Medicine, University of Texas MD Anderson Cancer Center, Houston, TX 77030, United States; Department of Bioinformatics & Systems Medicine, McWilliams School of Biomedical Informatics, University of Texas Health Science Center at Houston, Houston, TX 77030, United States; Department of Electrical and Computer Engineering, University of Virginia, Charlottesville, VA 22903, United States; Department of Bioinformatics & Systems Medicine, McWilliams School of Biomedical Informatics, University of Texas Health Science Center at Houston, Houston, TX 77030, United States

**Keywords:** Cell type annotation, scRNA-Seq, Artificial Intelligence, Pathway informed transformer, Deep learning

## Abstract

**Motivation:**

Single-cell RNA sequencing (scRNA-Seq) technology enables detailed exploration of gene expression at the individual cell level, crucial for annotating cell types and understanding cellular diversity. Traditional methods for cell type annotation often rely on marker genes and manual labeling, posing challenges due to low data quality and incomplete reference datasets.

**Results:**

We developed CeLLTra, a novel contrastive learning framework that leverages a Transformer-based model integrating biological pathway information to group genes into super tokens, effectively capturing comprehensive gene expression from scRNA-Seq data. By combining this pathway-informed Transformer with a pretrained domain-specific language model, CeLLTra accurately aligns cell-type annotations with gene expression profiles. Evaluations on a large-scale human scRNA-Seq dataset showed that CeLLTra significantly outperformed state-of-the-art methods in supervised and zero-shot cell-type prediction. Additionally, CeLLTra generalized well to external datasets, improving clustering performance and enabling better characterization of cancerous cell states in tumor-infiltrating myeloid cells from non-small cell lung cancer patients.

**Availability and implementation:**

CeLLTra is freely available on GitHub (https://github.com/WJZheng-group/CeLLTra) and Zenodo (https://doi.org/10.5281/zenodo.17666735). The datasets underlying this article are the following: GSE201333 and GSE127465. All these datasets are publicly available and can be freely accessed on the Gene Expression Omnibus repository.

## 1 Introduction

Single-cell RNA sequencing (scRNA-Seq) technology enables the exploration of gene expression patterns at the individual cell level (Tabula Muris Consortium *et al.* 2018, Tabula Sapiens Consortium *et al.* 2022, Yao *et al.* 2023, Gabitto *et al.* 2024). This detailed view allows for the annotation of cell types, facilitating the understanding of cellular diversity and its changes under various conditions (Svensson *et al.* 2018, Chen *et al.* 2019, Perez *et al.* 2022, Jin *et al.* 2025). Annotating cell types using scRNA-Seq data involves clustering cells with similar gene expression profiles and assigning them to known cell types or identifying new categories (Wang *et al.* 2021, Yang *et al.* 2022, Hou and Ji 2024). Despite its importance for downstream analyses, accurate cell type annotation remains challenging due to low data quality, cellular heterogeneity, and the lack of comprehensive reference datasets (Kiselev *et al.* 2019, Fischer *et al.* 2024).

Several existing approaches utilize marker genes for cell type annotation (Pliner *et al.* 2019, Zhang *et al.* 2019, Grabski and Irizarry 2022). Typically, unsupervised clustering is conducted first, grouping individual cells based on expression profile similarities. Once clustered, cells are annotated based on the expression of known marker genes for each cell type (Zeisel *et al.* 2015, Kiselev *et al.* 2019, Luecken and Theis 2019, Zilionis *et al.* 2019). This process involves identifying marker genes uniquely expressed in specific cell types and using this information for annotation. Marker genes can be identified by comparing scRNA-Seq data across different cell types or using existing knowledge about gene expression in particular cell types (Campbell and Yau 2019, Guo and Li 2021). The expression of these marker genes in individual cells facilitates their assignment to specific cell types.

However, annotation methods relying on existing cell markers often suffer from the lack of curated marker gene lists, which limits their generalizability and robustness (Wang *et al.* 2021). To address this limitation, supervised machine learning methods have been explored. These methods learn transferable gene expression patterns from labeled scRNA-Seq data to annotate cell types in new datasets (Yang *et al.* 2022). While supervised machine learning can achieve high accuracy in predicting known cell types, it struggles to generalize to novel cell types without prior training data. Annotating unseen cell types in scRNA-Seq data is beneficial as it reduces the need for curated marker genes for different cell types (Wang *et al.* 2021, Yixuan *et al.* 2023). However, this task poses significant challenges for both unsupervised clustering and supervised classification methods (Kiselev *et al.* 2019, Luecken and Theis 2019, Godec *et al.* 2022). Marker genes for unseen cell types may not be well-defined or previously characterized, making it difficult for clustering methods to label cell clusters. Additionally, supervised methods lack reference or training datasets to annotate these unseen cell types.

To address these challenges, OnClass was developed to annotate unseen cell types by leveraging the controlled vocabulary of cell ontology (Beisswanger *et al.* 2008, Diehl *et al.* 2016, Wang *et al.* 2021). OnClass learns the relationship between known and unknown cell types using the semantic meanings and hierarchical structure of cell ontology. However, during OnClass training, cell names are expressed in natural language (e.g., Müller cell) within training datasets, which must be mapped to controlled cell types in the cell ontology. The mapping procedure can introduce inconsistencies in cell annotations and limit the use of large-scale scRNA-Seq datasets. Moreover, the quality and completeness of the cell ontology remain bottlenecks for the performance of OnClass.

Recently, scRNA-Seq foundation models have emerged as promising tools to overcome these annotation challenges by learning extensive gene-expression relationships from large-scale datasets (Yang *et al.* 2022, Fischer *et al.* 2024, Hao *et al.* 2024). Models like scGPT (Cui *et al.* 2024) and GeneFormer (Theodoris *et al.* 2023) utilize powerful Transformer architectures trained on millions of single cells, enabling them to capture complex gene expression contexts and relationships effectively. However, foundation models face significant computational challenges when processing the high-dimensional data typical of scRNA-Seq, which often includes expression profiles for tens of thousands of genes per cell. Importantly, a single gene rarely acts in isolation; instead, genes exert their biological functions through coordinated interactions within pathways (Ideker *et al.* 2001, Kitano 2002). Recognizing both the computational burden and the biological interdependence of genes, integrating pathway-level information has emerged as an effective strategy. For instance, PathFormer leverages pathway-based gene groupings to improve model efficiency and performance, demonstrating the value of pathway-centric representations in biological analysis (Liu *et al.* 2024).

In this study, we developed a novel Transformer-based model to extract gene expression patterns from individual cells, leveraging the capabilities of Transformers and large-scale available data. To address the issue of long sequences and effectively identify gene expression patterns, we used biological pathway information to group genes as inputs to the Transformer. Furthermore, we designed CeLLTra, a contrastive cell-name-gene-expression learning framework, to align cell names with gene expression patterns, utilizing a pretrained domain-specific language model to annotate cell types unseen in the training data. Our Transformer-based model outperformed the current state-of-the-art on a large human single-cell RNA-seq dataset. Additionally, the contrastive learning framework effectively predicted unseen cell types while maintaining high performance on known cell types. We also applied our model to cancer cell state classification, significantly improving the clustering performance of cancerous cell subtyping in scRNA-Seq data from lung cancer patients.

## 2 Materials and methods

### 2.1 Datasets

Tabula Sapiens is a comprehensive human scRNA-Seq dataset comprising data from 24 different tissues and organs, publicly available from GEO under accession ID GSE201333. After quality control, over 480 000 single cells were retained, representing more than 400 distinct cell types with reference transcriptome profiles (Tabula Sapiens Consortium *et al.* 2022). To process the raw scRNA-Seq data for model training and prediction, we first filtered out cells with fewer than 200 genes and genes expressed in less than 0.1% of the total number of cells. Next, we normalized each cell by scaling the total counts to 10 000, ensuring that every cell had the same total count after normalization. We then applied a logarithmic transformation to the counts for each cell, which served as input for our model. To assess the impact of gene numbers on cell type prediction, we also identified highly variable genes for model input. All preprocessing steps were conducted using scanpy (Wolf *et al.* 2018).

### 2.2 Biological pathway-informed gene expression transformer

**Figure 1.**
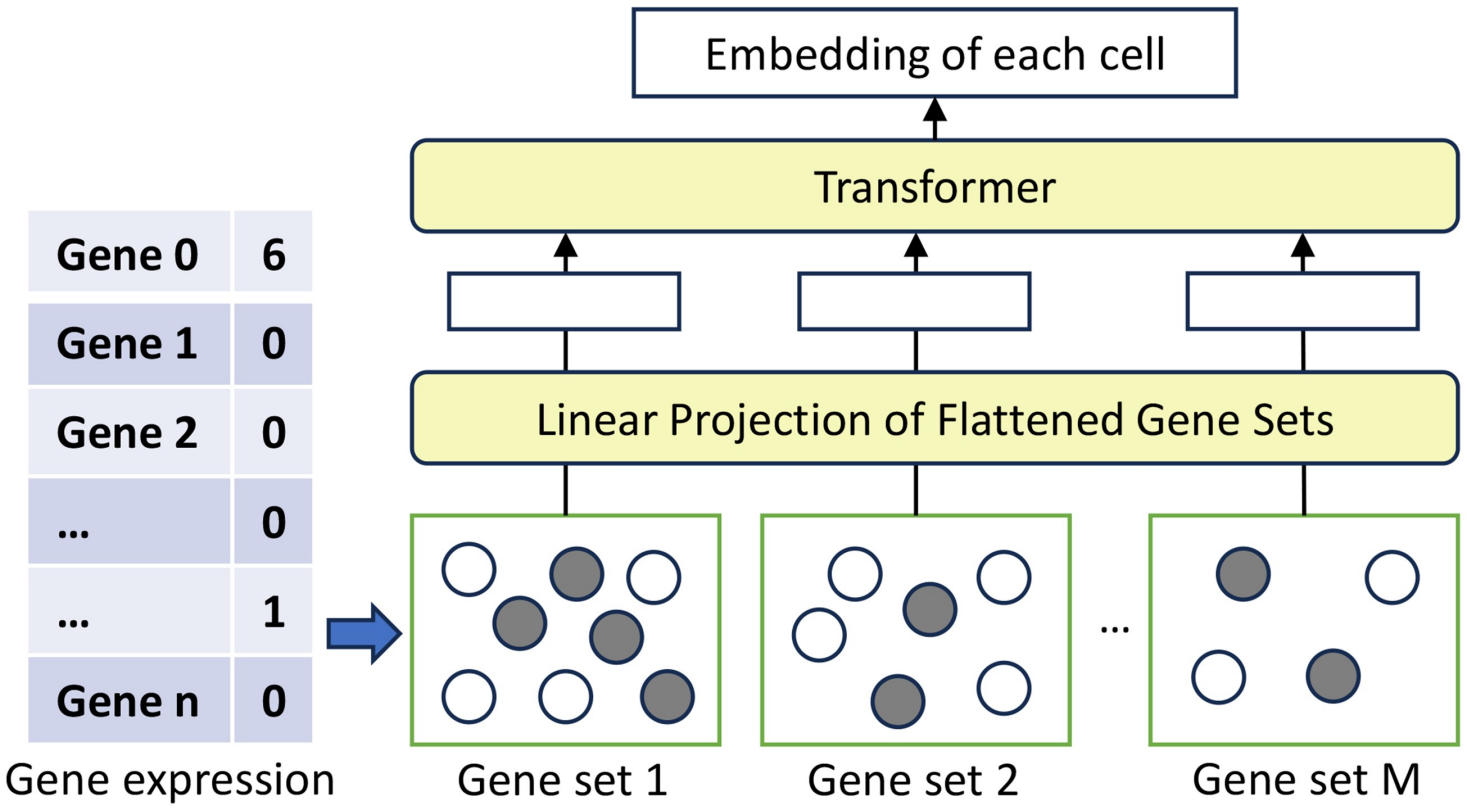
The architecture of pathway-informed gene expression Transformer model for representing the gene expression profile of each cell. The input for this model is a single cell’s gene expression profile, consisting of a list of genes and their corresponding expression values. Initially, we use pathway information to group these genes into multiple gene sets. Each gene set then serves as an input token to the Transformer model. The gray circle indicates that the gene expression value for a specific gene is 0. A linear projection layer is designed to learn the representation of each gene set.

Figure 1 shows the overview of the biological pathway-informed gene expression Transformer. A typical transcriptome profile contains thousands of genes, which can cause the long sequence issue (Yang *et al.* 2022) due to the self-attention mechanism in Transformer (Bulatov *et al.* 2024). To prepare the input for gene expression, we group the gene list in each gene expression profile $x\in R^{n}$ into M gene sets, where *n* is the number of genes and M is the number of gene sets. The KEGG pathway is utilized to group the genes into gene sets (Kanehisa 2002). Since some genes do not have the pathway membership information, we randomly grouped these genes without pathway information into gene sets of fixed size. The gene sets would serve as the input tokens for the Transformer model for the representation learning of each cell (Dosovitskiy *et al.* 2020).

To learn the representation of each cell, we first need to learn an embedding $Z_{i}$ for each gene set. To achieve this, we add an embedding layer to represent the embedding $G_{j}\in R^{D}$ for each gene $g_{j}$ where D is the gene embedding dimension. Then, we integrate the expression value $x_{j}$ of $g_{j}$ into the corresponding gene embedding by elementwise multiplication $x_{j}G_{j}$. The gene set embedding $Z_{i}$ is calculated by averaging the embedding of all genes in this gene set:


$$Z_{i}=\frac{1}{n_{\mathrm{gene}}}\sum_{j=1}^{n_{\mathrm{gene}}}x_{j}G_{j}$$

where $n_{\mathrm{gene}}$ is the number of genes in this gene set.

Similar to BERT’s ‘[class]’ token, we also add a learnable embedding to the start of the input tokens, which can serve as the representation of this whole gene expression profile (Devlin *et al.* 2018). Notably, we do not use positional encoding for these gene sets since the orders of different gene sets do not imply any biological meaning in this setup.

### 2.3 Contrastive cell-name-gene-expression learning

**Figure 2.**
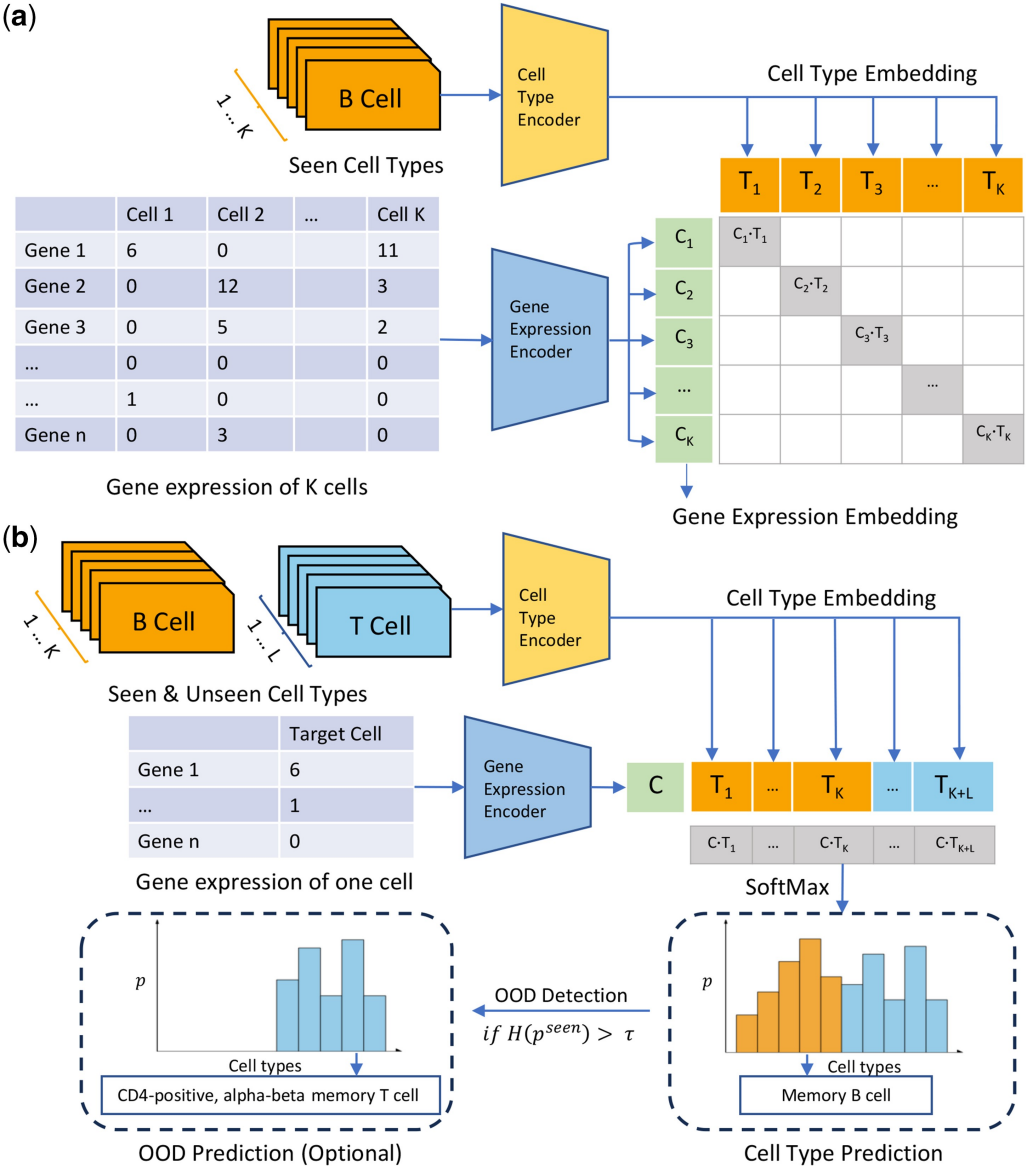
The framework of CeLLTra during pretraining and inference stages. (a) The pretraining stage of the proposed contrastive cell-name-gene-expression learning framework for aligning cell name with gene expression patterns. (b) Inference stage of CeLLTra to predict cell type for a given single-cell gene expression profile. OOD detection is consequently performed on the predicted probability of seen types.

In this section, we present the contrastive cell-name–gene-expression learning framework, CeLLTra, for zero-shot cell-type annotation. The core intuition is to align gene expression representations with cell names, leveraging the strong generalization capabilities of language models to infer unseen cell types using cell names as intermediaries (Radford *et al.* 2021). As illustrated in Fig. 2a, the pretraining stage aligns gene expression and cell name representations within a shared embedding space. Then, we employ batch-wise contrastive learning to establish these alignments. Each batch consists of K cells, where the gene expression encoder $E_{c}$ and the cell type encoder $E_{t}$ generate initial embeddings for the gene expression $c_{i}$ and cell name $t_{i}$ of each cell in the batch:


$$\forall 1\leq i\leq K,\;C_{i}=E_{c}(c_{i}),\;T_{i}=E_{t}(t_{i})$$

where the gene expression encoder $E_{c}$ is implemented using the gene expression Transformer described in the previous section, and the cell type encoder is $E_{t}$ based on PubMedBERT (Gu *et al.* 2021), a biomedical domain-specific pretrained language model. To align the representations, we train a linear projection layer that maps the cell name embeddings $T_{i}$ into the gene expression embedding space $C_{i}$. Within $C={\left\{C_{i}\right\}}_{i=1}^{K}$ and $T={\left\{T_{i}\right\}}_{i=1}^{K}$ in each training batch, we pair each gene expression representation with every cell name, forming a total of K×K gene-expression-cell-name embedding pairs. Among these, the matching pairs $\left\{C_{i},\;T_{i}\right\}$ (highlighted as blue diagonal elements in Fig. 2) represent the correct cell-type assignments, while the remaining pairs are treated as non-matching. The training objective is to maximize the cosine similarity for matching pairs while minimizing it for non-matching pairs using a cross-entropy loss function. Additionally, different cell types (names) are sampled in each batch to enhance pretraining effectiveness.

Figure 2b illustrates the inference stage of our proposed framework. Given a single-cell gene expression profile, the data is first processed by the gene expression encoder $E_{c}$ and the projection layer to generate a gene expression embedding C. Simultaneously, all cell type embeddings T for the candidate cell types (both seen and unseen) are obtained using the cell name encoder and projection layer. Next, similarity scores are computed between the gene expression embedding C and the cell name embeddings T, and the cell type with the highest similarity score is then assigned as the predicted label for the given cell.

### 2.4 Out-of-distribution detection for unseen cell type prediction

In the context of zero-shot cell type prediction, the training bias issue refers to the model’s tendency to favor seen cell types that are present in the training data, even when predicting on unseen types. This arises because the contrastive learning objective aligns gene expression only with seen cell type embeddings during training, leading the model to overfit familiar patterns of seen types. To address this issue, CeLLTra incorporates an entropy-based out-of-distribution detection strategy to improve the recognition of novel cell types (Fig. 2b). Specifically, we first calculate the entropy of the probability scores based on the seen cell types among all candidate cell types in the inference stage. If the entropy of the probability distribution on seen cell types exceeds a predefined threshold, the cell is classified as belonging to an unseen cell type category. The hypothesis is that cells with high entropy in their probability distribution from the prediction are less likely to belong to a well-characterized seen cell type and more likely to represent a novel or unseen cell type. This OOD detection enhances our model’s ability to accurately identify and classify unseen cell types, improving its robustness and generalizability in practical applications.

### 2.5 Baseline models

*scBERT—*scBERT is a Transformer-based single-cell cell-type annotation model (Yang *et al.* 2022). There are two major differences between our pathway-informed gene expression Transformer and scBERT. First, instead of using the continuous gene expression value as the input of the Transformer, scBERT discretizes the continuous expression variables into intervals and learns a representation for each interval. Secondly, scBERT employed a Transformer variant, Performer, to solve the long sequence issue. Specifically, Performer can handle the full gene list as input by changing the cross-attention calculation. Since scBERT cannot predict the unseen cell types in the training data without the reference dataset, we just benchmarked scBERT with our model in the supervised cell type prediction. For a fair comparison, we pretrained scBERT and pathway-informed gene expression Transformer on the same training data, and the evaluation results were obtained on the same test set.

*scGPT—*scGPT is a generative pretrained Transformer foundation model for single-cell biology (Cui *et al.* 2024). scGPT is trained over 33 million cells from 51 human tissues while leveraging masked-attention mechanisms to jointly learn cell and gene embeddings from non-sequential data. Through fine-tuning, scGPT can be adapted to diverse tasks such as cell type annotation and multi-omic integration. Similar to scBERT, we pretrain and test scGPT on the same dataset with CeLLTra.

*OnClass—*OnClass is an algorithm designed to annotate cell types in scRNA-Seq data that are not present in the training data (Wang *et al.* 2021). OnClass first runs a supervised cell type prediction model to generate a softmax output for the seen cell types in the training dataset. It then utilizes the cell ontology hierarchy to distribute the prediction probability of the training cell types to other nodes in the cell ontology using a random walk with restart algorithm. The prediction for the unseen cell type is obtained by selecting the cell type with the highest probability within the cell ontology. We compared OnClass with our contrastive cell-name-gene-expression learning model for both unseen and seen cell type prediction.

## 3 Results

The input gene number of every single cell after processing for the Tabula Sapiens dataset is 27 985. We use the KEGG pathway downloaded from (https://www.genome.jp/kegg/pathway.html) on 14 April 2022, to group genes into gene sets. There are 13 545 genes in scRNA-Seq data with pathway membership information, which can be grouped into 417 pathways. For the left 14 426 genes, we randomly split them into 132 gene sets with a size of 110. The gene embedding layer has 768 dimensions. The number of attention heads is 12. The pathway-informed gene expression Transformer has 12 Transformer layers. The intermediate linear layer in each Transformer layer has 3072 output features. The pretraining of the Pathway-Informed Transformer model used a learning rate of 5e−5 and a batch size of 32. For training CeLLTra, we projected the embedding of gene expression and cell type into a shared space with dimension 512 to compare the similarity. The learning rate is 1e−5 and the batch size is 32.

### 3.1 Biological pathway-informed gene expression transformer achieves superior performance on supervised cell type prediction

We split the Tabula Sapiens dataset into training, validation, and test sets with a ratio of 7:1:2 and trained and tested our pathway-informed gene expression Transformer model and scBERT for fair comparison. Table 1 shows the performance of these models under different conditions. First, using all gene expression values instead of just highly expressed genes can boost the performance of the model, leading the performance to an increase from 0.889 to 0.923. Both scBERT and scGPT pretrained on the same dataset underperformed our model. Meanwhile, replacing the pathway grouping with random gene set grouping caused inferior performance, with the F1 score decreasing from 0.923 to 0.895. We provide additional experimental results on alternative grouping approaches in the Supplementary Material, available as supplementary data at *Bioinformatics* online.

**Table 1.** The performance of pathway-informed gene expression transformer (CeLLTra^-^) and scBERT for supervised cell type prediction. Bold value indicates the best performance.

Method	Input size	Gene grouping	F1 score
CeLLTra	Genes in at least 0.1% cells expressed	Pathway	**0.923**
CeLLTra	Genes in at least 0.1% cells expressed	Random	0.895
CeLLTra	Highly variable genes	Pathway	0.889
scBERT	Genes in at least 0.1% cells expressed	–	0.890
scGPT	Highly variable genes	–	0.900

### 3.2 Contrastive cell-name-gene-expression learning for Zero-Shot cell type annotation

**Figure 3.**
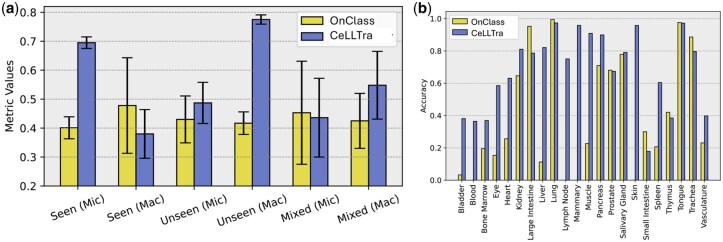
(a) The performance of OnClass and CeLLTra in the mixed test set with both seen and unseen cell types. We calculated both micro-F1 (Mic) score and weighted Macro-F1 (Mac) score. (b) The classification accuracy of different cell types in the test set.

To test the transferable prediction ability from seen cell types to unseen cell types, we randomly split the Tabula Sapiens dataset into training and test sets, ensuring that 10% of the cell types in the test set were unseen during training. We developed the model on the training set and evaluated its performance on seen, unseen, and mixed cell types in the test set. Considering the imbalanced cell numbers across different cell types, we calculated both Micro- and Weighted Macro-F1 scores. The F1 scores for seen and unseen cell types were calculated based on their ground truth labels separately, and the Mixed F1 score was computed as the harmonic mean of the Seen and Unseen F1 scores (Fig. 3).

**Figure 4.**
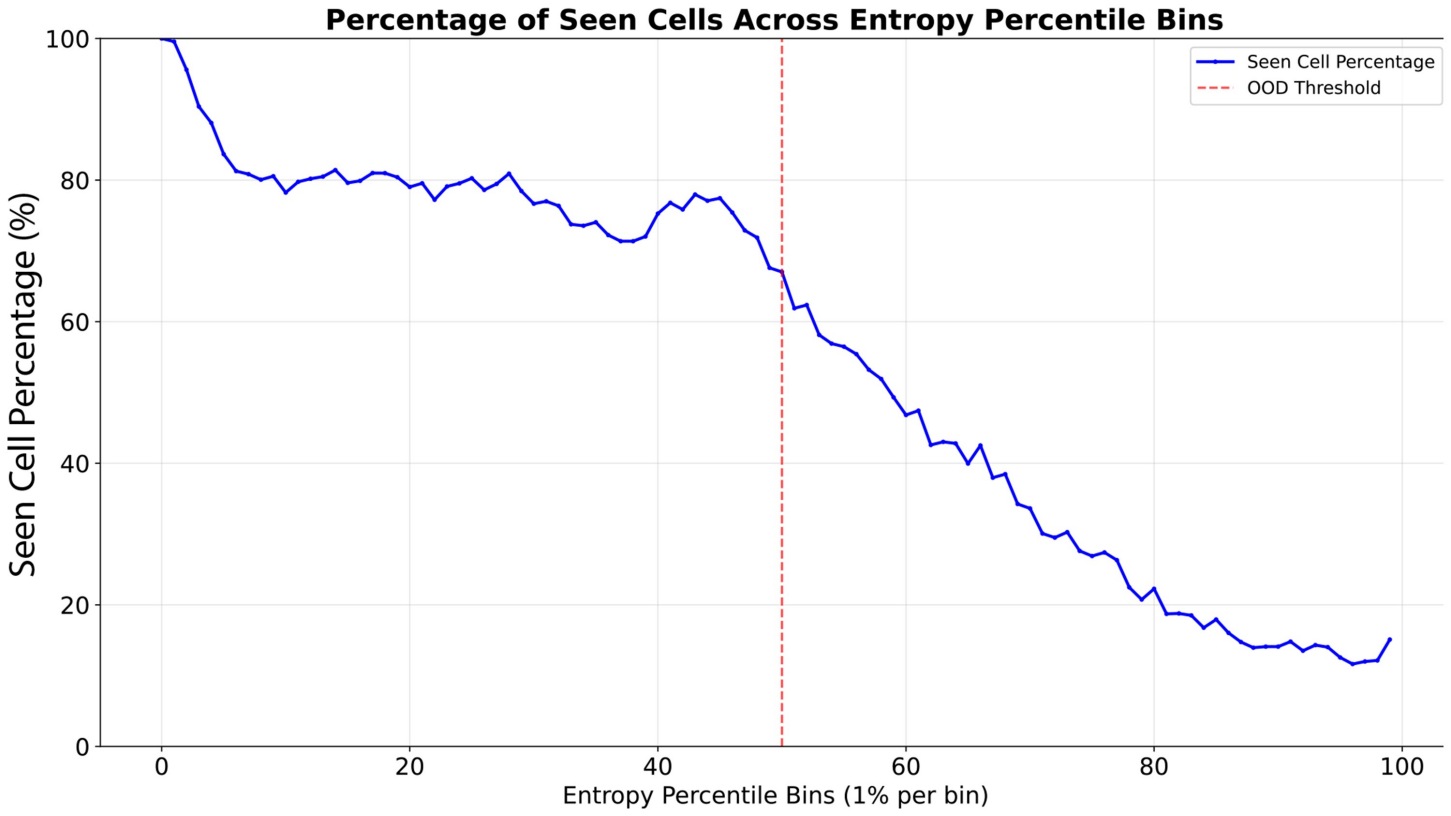
The correlation between the proportion of seen cell types and entropy, we first sort the predictions of all cells in ascending order with respect to entropy. then evenly partition the predictions into 100 bins. For each bin, we compute the proportion of seen cell types and visualize the results as a line chart.

We provided all cell types in this dataset as candidate cell types and predicted the final cell types for evaluation. We incorporated OOD detection after the inference stage of CeLLTra since the test set contains cells from both the seen and unseen cell types. We use the second quartile of all the predicted values in the test set as the thresholds for OOD detection. Additionally, to systematically evaluate the impact of different selections of unseen cell types on model performance, we conducted five rounds of experiments. In each round, we randomly selected 17 from the 136 cell types as unseen types (with the remaining 119 categories as seen types) to assess the model’s adaptability and robustness. To substantiate the claim that seen cell types are associated with lower prediction entropy, we examined and visualized their correlation, as illustrated in Fig. 4.

In both seen and unseen scenarios, CeLLTra exhibits lower variance, underscoring the stability inherent in our model design. The overall performance for both seen and unseen cell type prediction reached a mixed Macro-F1 score of 0.531 (Fig. 3a), considerably surpassing the OnClass prediction. These results indicate that our method improves cell type predictions for both seen and unseen cell types in mixed test data, even without prior knowledge of cell type visibility. Figure 3b shows the classification accuracy across different cell types in the test set. Notably, OnClass exhibits extreme performance variance, failing to annotate cell types entirely in certain organs. In contrast, CeLLTra not only achieves superior overall performance but also demonstrates greater stability across diverse organ types.

### 3.3 Generalizability and stability of Cell-Types annotation

**Figure 5.**
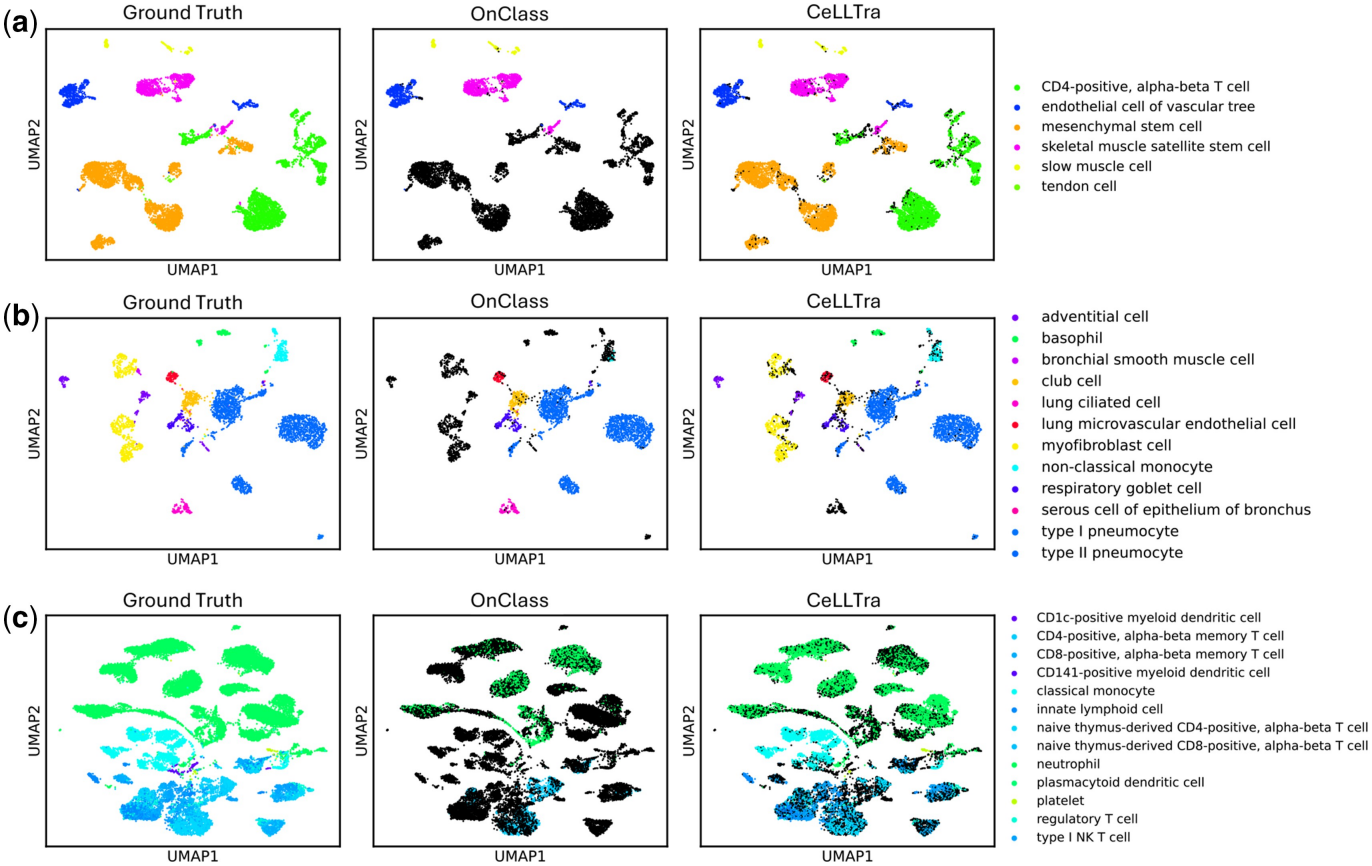
2-D UMAP visualization of cell type prediction for different organs muscle (a), lung (b), and spleen (c). The subfigure in each panel shows the annotation results of onclass (middle), our method (right), and ground-truth labels (left) for cells in this organ where black labels indicate wrong predictions.

To further assess whether CeLLTra maintains consistent performance across different cell types or exhibits extreme inaccuracies for specific ones, we visualize cell type annotations in Fig. 5a–c and Figs 1–4, available as supplementary data at *Bioinformatics* online. To provide a more intuitive and detailed comparison, we plot the UMAP of cell expression profiles from each specific organ in individual subfigures. Each UMAP is colored to reflect either the ground-truth cell types or the predicted cell types generated by the corresponding method (OnClass or CeLLTra). Misclassified predictions are additionally highlighted in black for clearer distinction. As shown in the UMAP visualization of OnClass, the presence of multiple large clusters of black points (representing incorrect predictions) indicates that its annotation accuracy for certain cell types is nearly negligible. In contrast, CeLLTra displays incorrect predictions only as isolated instances across all organ-specific cells, highlighting its robustness and superior generalizability in maintaining accurate annotations across diverse cell types.

### 3.4 Tumor-infiltrating myeloid cells clustering

We test the generalizability of the prediction of CeLLTra on a tumor-infiltrating myeloid cells prediction task. The data were downloaded from GEO using accession ID GSE127465 (Zilionis *et al.* 2019). This dataset contains 54 773 single cells from non-small cell lung tumor and the blood of 7 patients. For each cell, there is an LM22 cell type annotation, which represents the tumor-associated myeloid cell states and is very important for understanding cancer regulators and immunotherapy targets. We used the prediction from OnClass and our contrastive learning model to augment the raw gene expression data for cell clustering. The clustering accuracy was calculated using the LM22 cell type annotation as ground truth. We concatenated the individually softmax-normalized model outputs and gene expression features and conducted K-Means (Pedregosa *et al.* 2011) and Leiden graph-clustering (Traag *et al.* 2019) for these 54 733 cells. We set the number of clusters in K-Means to 21 to match the number of LM22 cell types in this dataset. As shown in Table 2, combining the prediction of our model with raw data significantly improved the clustering performance from 0.418 to 0.451 for K-Means and 0.476 to 0.559 for Leiden clustering. The improvement of our model is much larger than that of the OnClass prediction, especially for the Leiden method. This result indicates that the predictions of our model contain useful information for tumor-infiltrating myeloid cells prediction, even if the model is trained on data from non-cancer samples.

**Table 2.** Clustering accuracy of single cells for LM22 cell types from NSCLC patients.

	Rawdata	w/prediction of on class	w/prediction of CeLLTra
Kmeans (*n* = 21)	0.418	0.436	0.451
Leiden graph-clustering method	0.476	0.495	0.559

## 4 Discussions

Cell type annotation is critical for various downstream tasks. Most existing methods require labeled data to train a model for cell type prediction. Unsupervised clustering-based methods need cell type-specific gene markers and manual labeling of the clustered results. As the generation of scRNA-Seq data accelerates, annotating unseen cell types is becoming increasingly necessary. Although OnClass can predict cell types within the cell ontology, this approach is limited by the ontology’s scope, especially for special conditions such as cancerous cell states. Our model addresses these limitations by utilizing a domain-specific pretrained language model to extract representations of any given cell type and calculate an alignment score for the gene expression data. This approach enhances the ability to annotate both seen and unseen cell types, providing more flexibility and accuracy in cell type prediction.

The pathway-informed Transformer helps extract patterns closely related to cell types. Grouping genes using pathways eliminates the long-sequence issue in Transformer models due to the cross-attention mechanism. Additionally, cell signaling pathways play critical roles in controlling cell functions (Davis and Ragan 2013). This is why our pathway-informed Transformer achieves superior performance in cell type prediction with high efficiency. However, the pathway database is still incomplete. More than half of the genes in the data lack pathway membership information. To address this, we randomly grouped these genes without pathway information into different gene sets as input tokens. Future work could explore integrating existing biological pathway and gene set databases, such as MSigDB (Liberzon *et al.* 2011), to generate fine-grained gene sets for Transformer input tokens.

The contrastive cell-name-gene-expression framework can help identify tumor-infiltrating myeloid cells in lung cancer patients. By concatenating the output of the contrastive learning framework with single-cell gene expression data for clustering cancerous cell states, we demonstrated improved clustering accuracy compared to using gene expression data alone. This indicates that the patterns learned from the training data contain generalizable features useful for related tasks. Labeled data provides additional information for clustering unseen and out-of-distribution data. With this insight, we can collect more scRNA-Seq data and scale up our model to train a large cell-name-gene-expression contrastive model for various downstream tasks. One benefit of our model is that it allows the use of any cell types expressed in natural language rather than relying solely on cell ontology, providing greater flexibility to incorporate diverse datasets for training large-scale models. Moreover, zero-shot prediction of unseen cell types relies on an efficient cell type encoder. The domain-specific pretrained model may not always extract useful information using cell names alone. Therefore, continuous improvement and validation of the cell type encoder using more cell name information is crucial to enhance prediction accuracy.

## 5 Conclusions

In this study, we developed a Transformer-based model to extract gene expression patterns from individual cells. To address the long sequence issue of the transformer architecture and effectively identify gene expression patterns, we used biological pathway information to group genes as super tokens to the Transformer. This approach enables the efficient inclusion of all sequenced genes, rather than a limited subset, for downstream analysis in scRNA-Seq data. Additionally, we designed CeLLTra, a contrastive cell-name-gene-expression learning framework, to align cell names with gene expression patterns using a pretrained domain-specific language model. Our pathway-informed Transformer-based model outperformed the current state-of-the-art on human single-cell RNA-seq datasets. The contrastive cell-name-gene-expression learning framework was also effective in predicting unseen cell types while maintaining performance on seen cell types. Furthermore, we applied our method to predict tumor-infiltrating myeloid cells in NSCLC patients, significantly boosting clustering performance. Our study highlights the great potential of large-scale cross-modality scRNA-Seq research. The pathway-informed Transformer can also be applied to other transcriptomics data, such as microarray and bulk RNA-Seq.

## Supplementary Material

btaf655_Supplementary_Data
