# Supplementary material for: CeLLTra: aligning cell names with gene expression via a pathway-informed transformer
Source: Bioinformatics. 2025 Dec 5;42(2):btaf655. doi: 10.1093/bioinformatics/btaf655 (PMC12881829; doi:10.1093/bioinformatics/btaf655)
Supplement: btaf655_Supplementary_Data [file btaf655_supplementary_data.pdf]

## 1. Alternative Grouping Approaches

In this section, we further analyze the performance of additional gene grouping methods, which encompass the following categories: ***(i)*** ***omit:*** We restrict the grouping to the genes included in the KEGG dataset and discard those that do not appear within it. ***(ii)*** ***coexp:*** For genes absent from KEGG, we group based on their coexpression patterns within the train data set. ***(iii)*** ***reactome:*** In addition to KEGG, we integrate the grouping information from Reactome, while genes not covered by either resource are randomly assigned to auxiliary groups. Beyond the aforementioned grouping methods, we also evaluate the ability of the attention layer (***attn***) to aggregate gene embeddings within each group. In the main text, our approach relied on simple embedding averaging. Based on the empirical results reported in Table S1, all variations of CeLLTra exhibit varying degrees of performance degradation compared to the original method, demonstrating the effectiveness of CeLLTra.

| Method | Micro F1 |
|---|---|
| CeLLTra+omit | 89.85 |
| CeLLTra+coexp | 89.89 |
| CeLLTra+reactome | 91.33 |
| **CeLLTra** | **92.30** |
| CeLLTra+attn | 89.68 |

**Table S1. Comparison of grouping strategies. All values are persentages**

## 2. UMAP Visualization

In this section, we provide the UMAP visualizations (Figure S1, S2, S3, S4) of the cell type annotation results w.r.t the organs that were not presented in the main text.

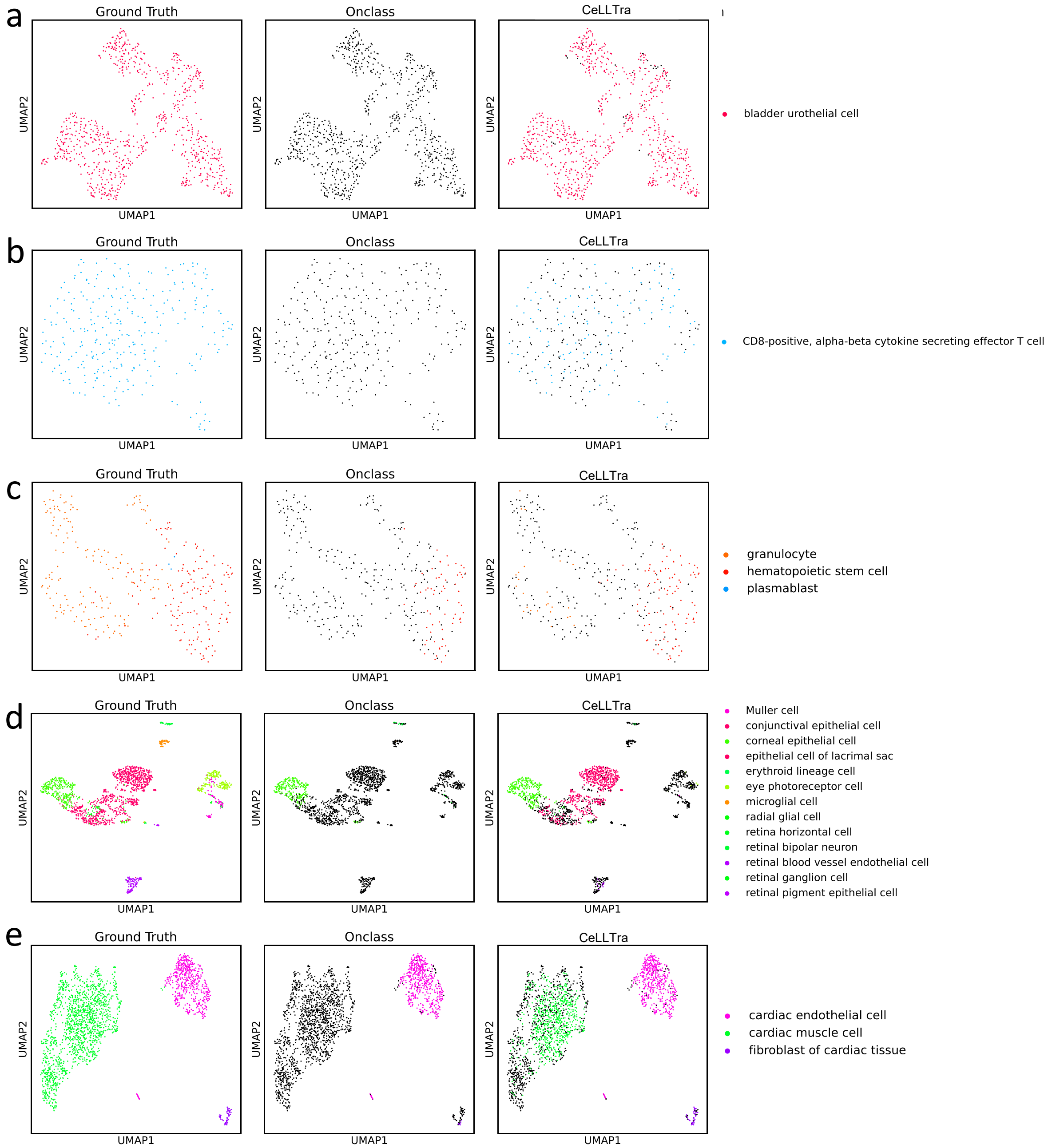


**Fig. S1.** UMAP visualization of annotation for cells in the bladder (**a**), blood (**b**), bone marrow (**c**), eye (**d**), and heart (**e**). Wrong cell-type predictions of the baseline model (Onclass) and our approach (PathAligner) are colored black.

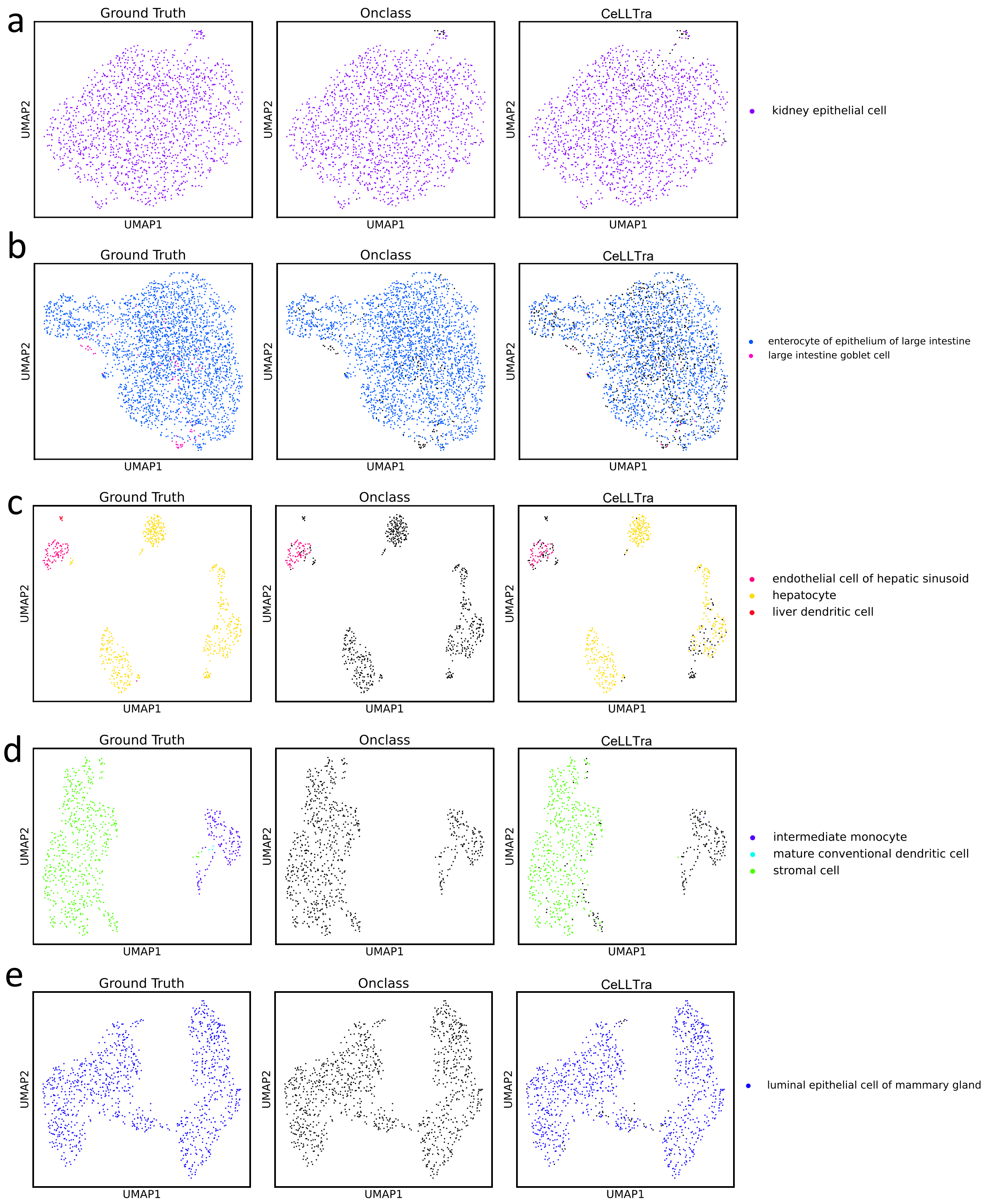


**Fig. S2.** Umap visualization of annotation for cells in kidney (**a**), large intestine (**b**), liver (**c**), lymph node (**d**), and mammary (**e**). Wrong cell-type predictions of the baseline model (Onclass) and our approach (PathAligner) are colored black.

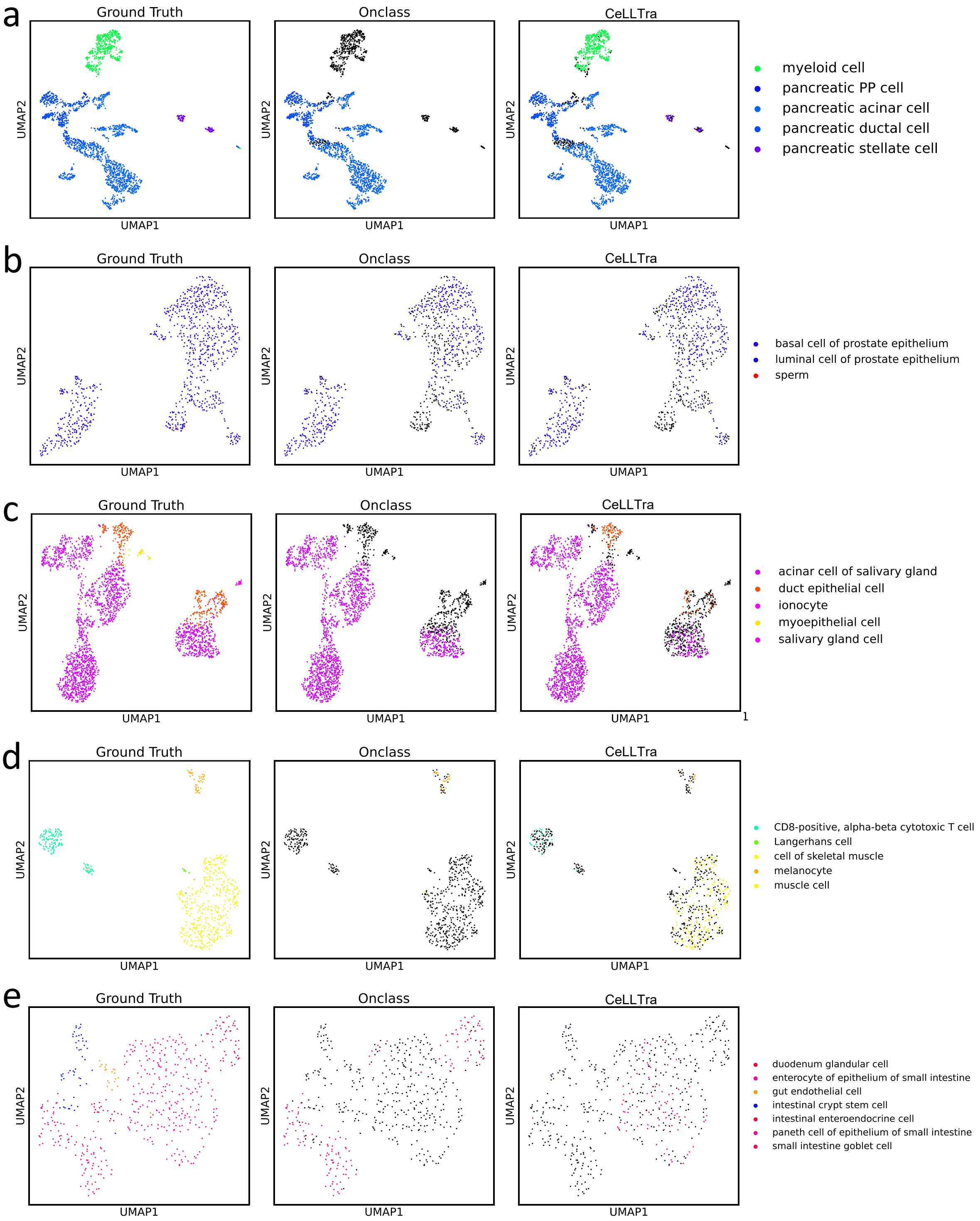


**Fig. S3.** Umap visualization of cells in the pancreas (**a**), prostate (**b**), salivary gland (**c**), skin (**d**), and small intestine (**e**). Wrong cell-type predictions of the baseline model (Onclass) and our approach (PathAligner) are colored black.

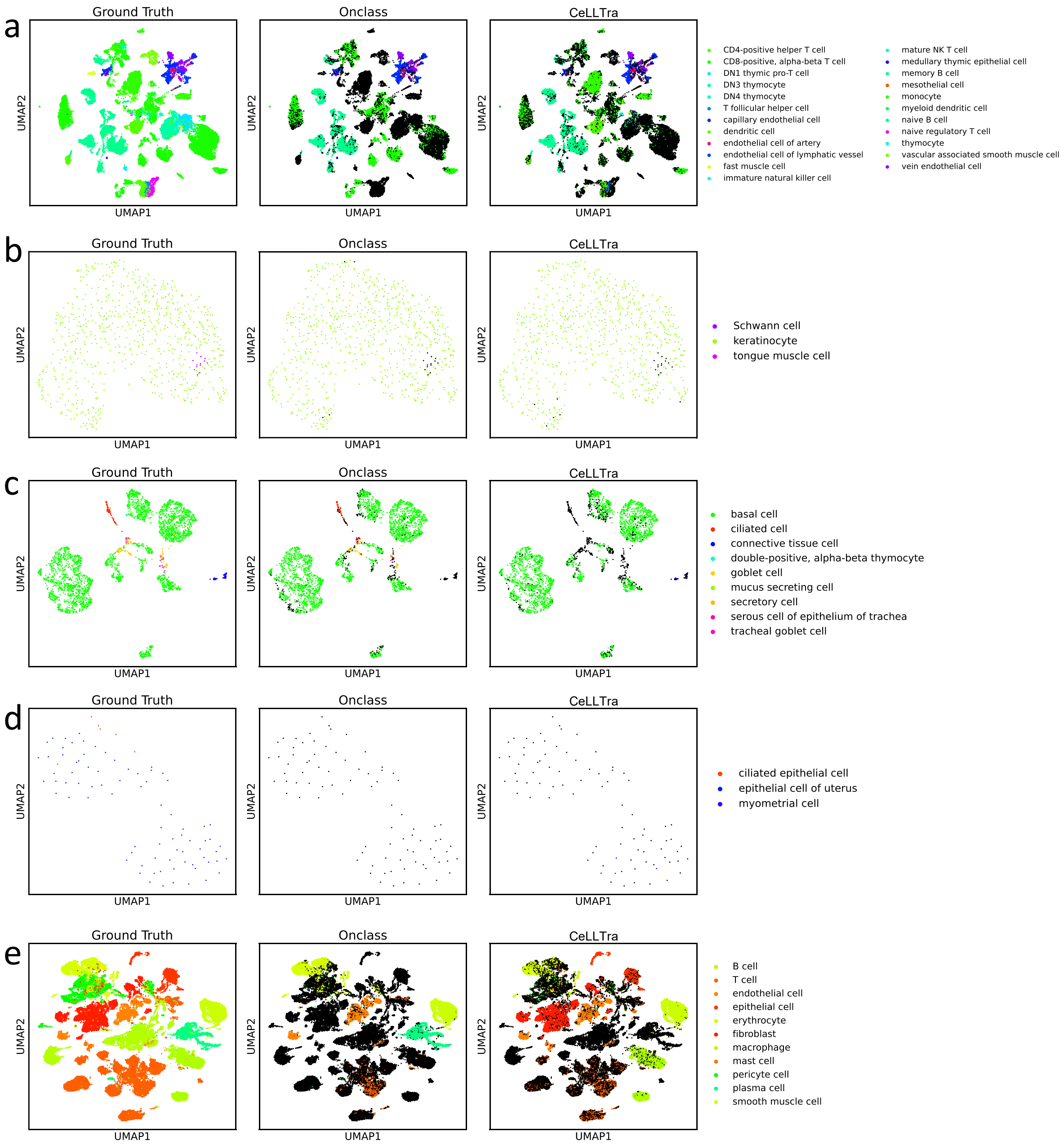


**Fig. S4.** Umap visualization of annotation for annotation for cells in thymus (**a**), tongue (**b**), trachea (**c**), uterus (**d**), and vasculature (**e**). Wrong cell-type predictions of the baseline model (Onclass) and our approach (PathAligner) are colored black.
